# Non-Steroidal Anti-Inflammatory Drugs Loaded to Micelles for the Modulation of Their Water Solubility

**DOI:** 10.3390/ijms242015152

**Published:** 2023-10-13

**Authors:** Christina N. Banti, Angelos G. Kalampounias, Sotiris K. Hadjikakou

**Affiliations:** 1 Inorganic Chemistry Laboratory, Department of Chemistry, University of Ioannina, 45110 Ioannina, Greece; cbanti@uoi.gr; 2 Physical Chemistry Laboratory, Department of Chemistry, University of Ioannina, 45110 Ioannina, Greece; 3 Institute of Materials Science and Computing, University Research Center of Ioannina (URCI), 45110 Ioannina, Greece

**Keywords:** anti-inflammatory drugs, aspirin, water solubility, delivery system development, micelles, sodium lauryl sulfate

## Abstract

The low water solubility of aspirin (ASPH) is well known, creating research challenges regarding both its composition and its delivery. Therefore, the development of new aspirin-based formulations that are water soluble is a research, technological, and financial issue. With the aim to improve the water solubility of ASPH, the micelle of formula **SLS@ASPH** (SLS = Sodium Lauryl Sulfate) was formed. The Critical Micelle Concentration (CMC) of SLS in the presence of ASPH was determined by ultrasonic velocity, complementary, and transient birefringence measurements. The **SLS@ASPH** was characterized by the melting point (m.p.), attenuated total reflection spectroscopy (FT-IR-ATR), and X-ray fluorescence spectroscopy (XRF) in a solid state and in a solution by ultraviolet-visible (UV-Vis) and ^1^H NMR spectroscopies. The SLS/ASPH molar ratio was determined to be 5/1 in **SLS@ASPH**. The inhibitory activity of **SLS@ASPH** towards lipoxygenase (LOX), an enzyme that takes part in the inflammation mechanism, was studied. The inhibitory activity of **SLS@ASPH** against LOX is 3.5-fold stronger than that of free SLS. The in vitro toxicity of the **SLS@ASPH** was tested on immortalized human keratinocyte (HaCaT) cells.

## 1. Introduction

The low water solubility of aspirin (ASPH) is well known, creating research challenges in terms of both its composition and its delivery [1]. By improving the water solubility of aspirin, the enhancement of its bioavailability and therapeutic efficacy is expected. This issue has been extensively studied, and various strategies have been explored to enhance the aqueous solubility of aspirin, such as particle size reduction, usage of solubilizing agents, and formulation techniques [2]. The reduction in particle size increases the surface area available for dissolution, thereby improving the water solubility of aspirin. Techniques such as micronization, nanosizing, or milling can be employed to achieve particle size reduction [3]. Converting aspirin into a salt form can improve its solubility in water. Salts such as aspirin lysinate or aspirin citrate have been developed to enhance the water solubility and dissolution characteristics of aspirin [4]. Finally, solubilizing agents are used to enhance the solubility of aspirin by forming inclusion complexes or micelles with the drug. Examples of solubilizing agents include cyclodextrins, surfactants, and co-solvents [5].

Micelles reduce the effective doses of drugs, and they protect the drugs within their core from the outer environment [6]. Sodium lauryl sulfate or sodium dodecyl sulfate (SLS) has been classified as readily biodegradable with no concern to human health by the United Nations Environment Program (UNEP) [7]. Moreover, sodium lauryl sulfate (SLS) is a widely used surfactant in cleansing products, cosmetics at doses ranging from 0.01 to 50%, and personal care products [8]. The structure of SLS simulates the phospholipid residues of membranes, which allows its penetration into the cytoplasm [9]. Consequently, its micelles can be disassembled in the cell membrane, releasing their ingredients [9].

**Scheme 1:**
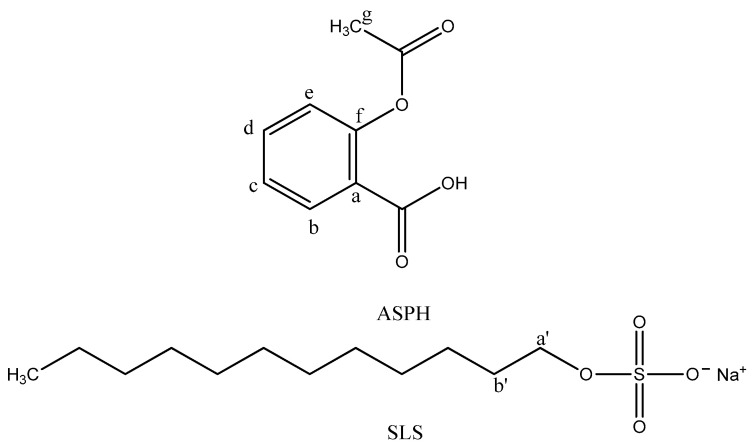
Molecular formulae of the **SLS@ASPH** ingredients **ASPH** and **SLS**.

In the course of our studies towards the development of drug delivery systems [10,11,12,13], the water-soluble micelle **SLS@ASPH** (ASPH = aspirin, SLS = Sodium Lauryl Sulfate (Scheme 1)) was formed and characterized by m.p., FT-IR-ATR, XRF, UV-Vis, and ^1^H NMR spectroscopies. Moreover, the CMC was determined by ultrasonic velocity, complementary, and transient birefringence measurements. The inhibitory activity of **SLS@ASPH** towards lipoxygenase (LOX) was studied. The in vitro toxicity of **SLS@ASPH** was tested against HaCaT cells. The SLS was chosen for the solubilization of ASPH since (i) it is used for drug delivery [10,11,12,13], (ii) SLS is used as an anionic body detergent and cleanser ingredient (in shower gels and toothpastes) without any harmful irritation [14], and (iii) the U.S. Food and Drug Administration (USFDA) recognizes it as a harmless food ingredient (21 CFR 172,822).

## 2. Results

### 2.1. Preparation and Characterization of Micelle

**Scheme 2:**
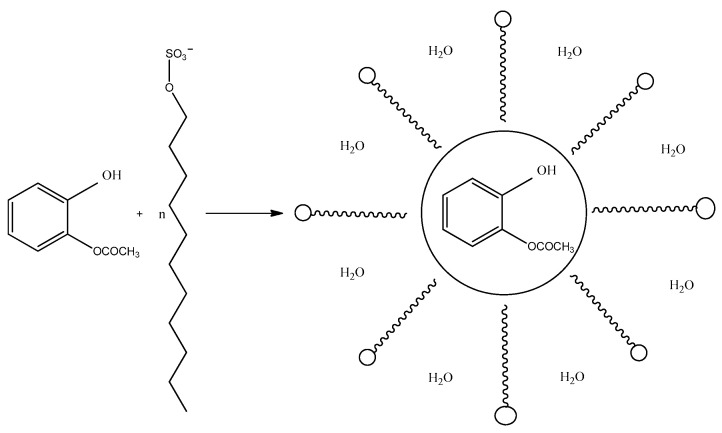
**SLS@ASPH** micelle self-assembly.

In order to increase the water solubility of ASPH, the micelles of the formula **SLS@ASPH** were characterized. Micelles are supramolecular assemblies that are formed in a liquid, in which surfactant molecules, like SLS, are dispersed. SLS molecules are made up of a hydrophilic head and a hydrophobic tail. When the concentration of the SLS increases above the CMC, it starts to associate in order to exhibit minimal water contact. This association turns the hydrophilic heads of SLS to be in contact with surrounding water molecules of the solvent, thereby impounding the hydrophobic tails towards the micelle center. The concentration for the formation of micelles (CMC) by free SLS is 8.3 mM [12]. Upon the addition of the drug ASPH during micelle assembly, the ASPH molecules are included in the lipophilic center of the SLS micelles (Scheme 2). The aggregatoion 

*(a) Determination of critical micelle concentration (CMC*) by *solutions density measurements:* For these measurements, the solutions of SLS and ASPH dissolved in triply distilled water and methanol, respectively, were prepared gravimetrically.

**Figure 1:**
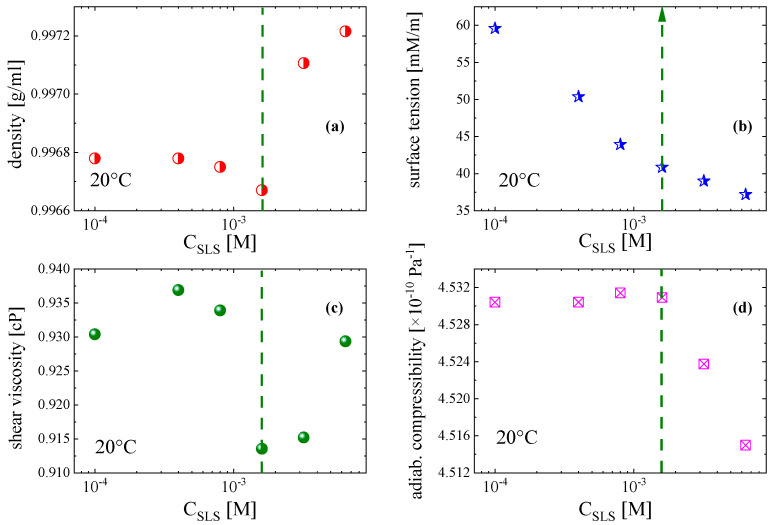
Density *ρ* (**a**), surface tension *γ* (**b**), shear viscosity *n_s_* (**c**), and adiabatic compressibility *κ_s_* (**d**) of all solutions prepared at 20 °C under isobaric conditions. The dashed lines show the inflection points that correspond to the CMC. The concentration of ASPH was constant and equal to 10^−4^ M for all solutions. Both SLS and ASPH were dissolved in triply distilled water and methanol respectively.

The density *ρ*, surface tension *γ*, shear viscosity *n_s_*, and adiabatic compressibility *κ_s_* data are presented in Figure 1. In all cases, the plots exhibit an inflection point when a certain concentration is reached. This well-defined concentration is associated with the micellization process and defines the CMC, which corresponds to [SLS] = 1.6 mM (or 0.05% *w*/*w*). All physical properties agree with each other in this view.

**Figure 2:**
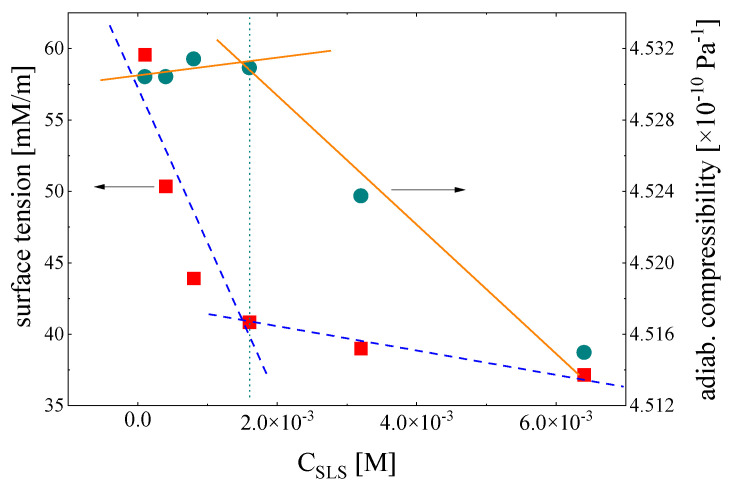
The surface tension (left axis) (red squares, blue line) and isentropic compressibility (right axis) (green circles, red line) are plotted against surfactant concentration at 20 °C under ambient pressure in a different representation.

Figure 1 shows several physical properties, three of which exhibit a sudden change in SLS concentration that signifies the CMC of the **SLS@ASPH**. In order to facilitate the observation of the CMC, the surface tension (left axis) and isentropic compressibility (right axis) are plotted against the surfactant concentration at 20 °C under ambient pressure in a different representation (Figure 2). Upon the micelles’ formation, a change in the slopes is observed. The dotted line denotes the critical micelle concentration.

*(b) Determination of CMC* via *ultrasonic velocity:* Ultrasonic velocity was measured by means of the pulse-echo technique [15,16].

**Figure 3:**
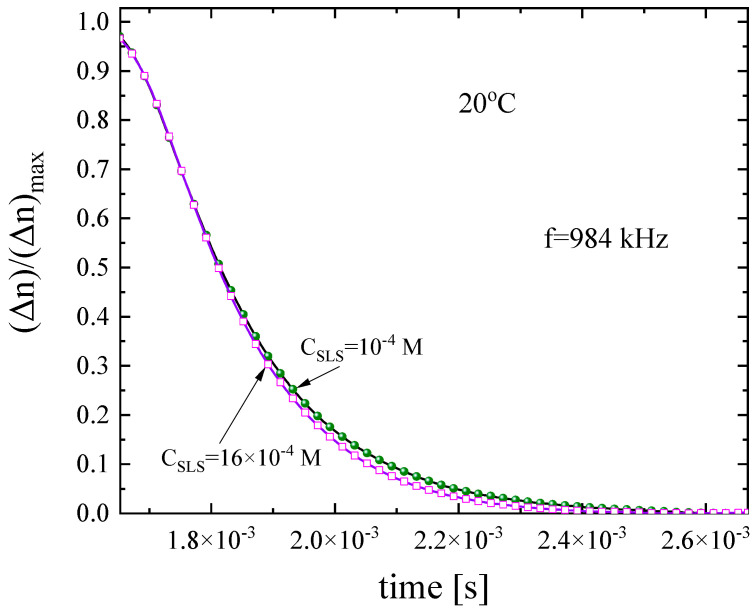
Representative fitting examples of the normalized birefringence signals in the field-off region corresponding to solutions with [SLS] = 0.1 mM and 1.6 mM. The concentration of ASPH is constant and equal to 10^−4^ M for both solutions. The working ultrasound frequency was 984 kHz, and the temperature was 20 °C. Symbols and solid lines correspond to experimental data and fitting curves, respectively. A smaller portion of the experimental points is presented for clarity. See text for details concerning the fitting procedure.

The analysis of the acoustically induced birefringence signals in the transient region allows one to progress in the comprehensive understanding of the involved dynamics and the geometrical properties of the system. Figure 3 shows representative ultrasonically induced birefringence signals for the S_1_ and S_4_ solutions.

The application of an external acoustic field into a liquid sample induces macroscopic anisotropy, and the system exhibits an optical retardation *δ*. The phase retardation is related to the intensity *I* of the light that passes through the analyzer as:

(1) $${sin}^{2}\left(\frac{\delta }{2}\right)=\left(\frac{I}{I_{0}}\right)$$

where *I*_0_ denotes the light intensity when the acoustic field is off, with the polarizer and the analyzer in parallel configuration. The intensity *I* is the intensity of the light “leakage” due to the birefringence and is measured with the analyzer in crossed polarization relative to the incident beam polarization. This polarizer-analyzer configuration ensures the absence of light transmission when the acoustic field is off.

The relation between birefringence and optical retardation is:

(2) $$\Delta n=\frac{\lambda \delta }{2\pi d}$$

In the above equation, *d* and *λ* denote the optical path length and the laser’s wavelength, respectively. 

The rise in the birefringence signal when the acoustic field is on is given by:

(3) $$\Delta n\left(t\right)={\Delta n}_{max}\left(1-e^{-{\left(t/\tau \right)}^{\beta }}\right)$$

The corresponding equation accounting for the birefringence decay when the acoustic field is off is:

(4) $$\Delta n\left(t\right)={\Delta n}_{max}e^{-{\left(t/\tau \right)}^{\beta }}$$

where Δ*n_max_* is the stationary or equivalently the steady-state value of the birefringence signal with all the species oriented parallel to the direction of the ultrasound propagation. *τ* is assigned the characteristic relaxation times for the orientation (rise region) and disorientation (decay region) processes, and parameter *β* is related to the polydispersion of the particles.

In Figure 3, two representative examples of the fitting procedure using Equation (4) corresponding to the decay region of the birefringence signal are shown. Pure liquids and mixtures composed of small molecules usually represent a relatively fast response [17]. Nevertheless, in the system studied in this work, the self-organization of SLS and the formation of larger supramolecular aggregates of the **SLS@ASPH** lead to a more perplexed structure, and the underlying dynamics are slower. The disorientation process through re-orientational diffusion is strongly affected by dipole–induced dipole interactions (DID interactions) between neighboring complex structural entities, and the timescale of the involved dynamics falls within the timescale of the ultrasonically induced birefringence technique.

In the field-off region, the birefringence signal is reduced due to orientational thermal fluctuations of the particles. The characteristic relaxation time *τ* of the disorientation process, as received from the fitting procedure, is associated with the hydrodynamic volume *V_h_* through the equation [18]

(5) $$\tau =n_{s}V_{h}/k_{B}T$$

In the above equation, the other symbols have their usual meanings, namely, *T* is the temperature, *k_B_* is the Boltzmann constant, and *n_s_* is the shear viscosity of the solvent. 

**Figure 4:**
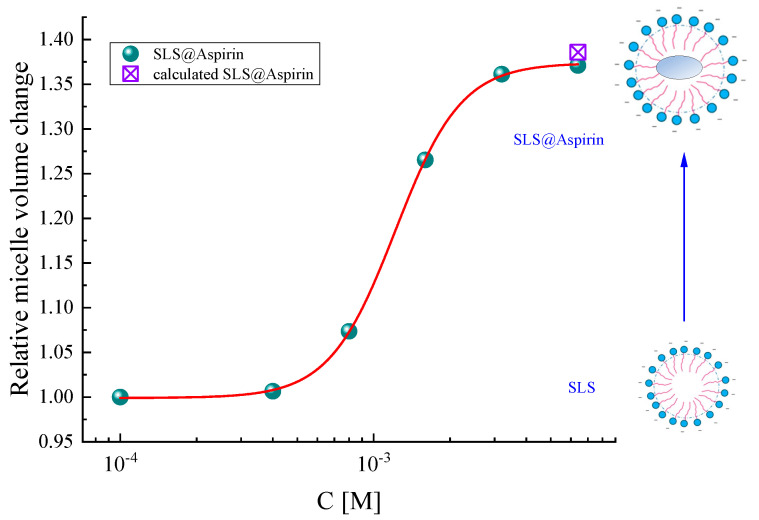
Relative volume change after the incorporation of aspirin into the SLS micelle as a function of the SLS concentration. Solid symbols correspond to experimentally estimated values, while square symbols designate the theoretically calculated volume change for the **SLS@ASPH** micelle. See text for more details concerning the theoretical volume evaluation of the SLS and **SLS@ASPH** micelles.

The hydrodynamic volume was estimated by applying the so-called Stokes–Einstein equation (Equation (5)), and the results were compared with the theoretically estimated volume and clearly indicated the incorporation of ASPH in the parental micelle. Subsequently, from the obtained hydrodynamic volumes, we estimated the relative volume change as a function of SLS concentration, and the results are presented in Figure 4. Figure 4 also shows the theoretically predicted volume change obtained from the initial volume of the SLS micelle and the final volume of the complex **SLS@ASPH** micelle. For the SLS micelle, we estimated the corresponding volume considering the micelle to be spherical with a radius equal to the size of the optimized one-dimensional structure of SLS. The volume of the **SLS@ASPH** micelle was estimated by considering the complex micelle to be an oblate spheroid. The major and the minor diameters of the aspirin, which was also considered to be a spheroid, were calculated following standard structure optimizing procedures in a vacuum environment without any interactions with the surroundings. Thus, the major diameter of the obtained **SLS@ASPH** micelle was considered the sum of two times the size of the SLS plus the major axis of the aspirin, while the minor diameter was estimated as the sum of two times the size of the SLS plus the minor axis of the aspirin. 

Therefore, as the ASPH molecule can be considered an oblate rather than a spherical molecule, and the SLS molecule is a linear (~17.7 Å) molecule, then the **SLS@ASPH** micelle will also be oblate with a major axis of 43.0 Å and minor axis of 37.7 Å. The volume of the SLS micelle was found to be 23.08 nm^3^, and the volume of the **SLS@ASPH** micelle was equal to 31.98 nm^3^. The relative volume change was 31.98/23.08 =1.38 (Figure 4). The relative volume change reached a plateau in terms of SLS concentration, which means that the size of the micelle remained almost constant within the experimental error (Figure 4).

The theoretically predicted and the experimental volume changes were in close agreement. A clear increasing trend was observed as a function of SLS concentration, revealing a clear volume change associated with the incorporation of aspirin into the SLS micelle, validating the proposed micellization mechanism. 

*(c) Determination of Aggregation number:* The aggregation number in the **SLS@ASPH** was found equal to 1.69. Details on the aggregation process and the aggregation number in the case of **SLS@ASPH** is given in the Figure S1 and supplementary (See SI file).

### 2.2. Preparation of Micelle

**Figure 5:**
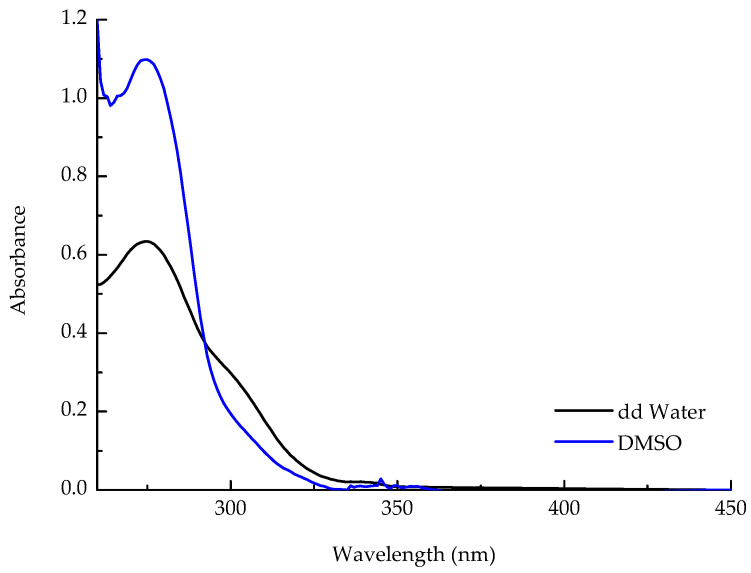
UV spectrum of **SLS@ASPH** in double distilled water (ddH_2_O) and in DMSO (1.2 mg/mL).

ASPH was suspended in water (1.3% *w*/*w*) at 37 °C. A water–SLS solution (9% *w*/*w*, which was higher than CMC (0.05%)) was consequently added to the previous one. The clear solution was filtered off, and the filtrate was slowly concentrated. Upon concentration of this solution, the **SLS@ASPH** was obtained. The solubility in water and in DMSO was determined by UV-Vis spectroscopy. Figure 5 shows the **SLS@ASPH** electronic spectrum in water and DMSO (1.2 mg/mL). The *w*/*w* % or mg/L concentration units were used rather than M because the molecular weight of the micelles could not be determined accurately.

### 2.3. Characterization of SLS@ASPH

*(a)*  *Solid state:*

**Figure 6:**
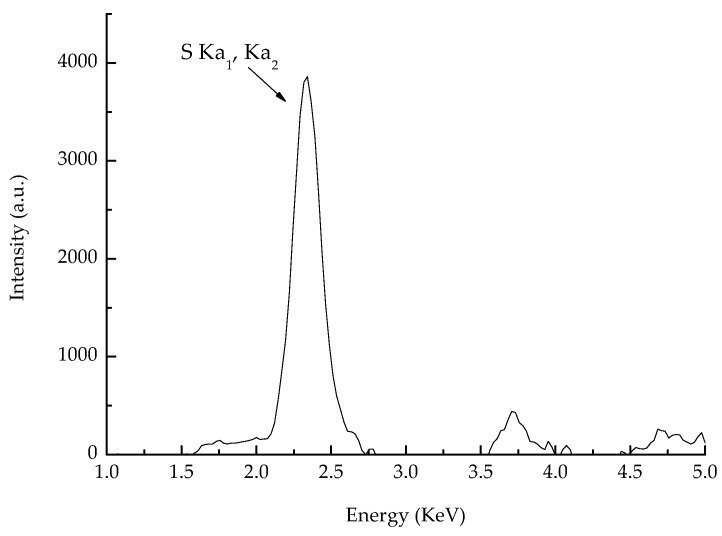
XRF spectra acquired from **SLS@ASPH**. S Kα X-ray emission was used for the quantitative determination of S.

*X-ray fluorescence spectroscopy of **SLS@ASPH***: The XRF spectrum of **SLS@ASPH** confirms the presence of S in the micelle and consequently the encapsulation of ASPH (Figure 6).

The content of sulfur was found to be 9.90% *w*/*w*, while the content of sulfur in pure SLS was 11.10% *w*/*w*. The calculated content of sulfur in **SLS@ASPH** was 9.86% *w*/*w* in the case of SLS/ASPH = 5:1. By accounting for the SLS/ASPH = 5:1 molar ratio, the content of ASPH in **SLS@ASPH** as calculated by XRF spectroscopy was 11.11% *w*/*w*.

**Figure 7:**
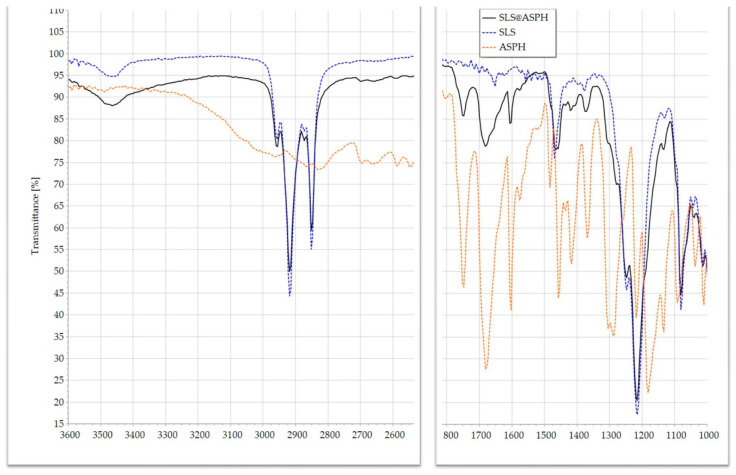
The ATR-FT-IR spectra of **SLS@ASPH**, ASPH, and SLS.

*Vibrational spectroscopy*: The vibrational bands at 2918 and 2851 cm^−1^ in the ATR-IR spectrum of **SLS@ASPH** are attributed to the v(C_aliphatic_-H) of SLS, indicating the presence of SLS in the micelle (Figure 7). The vibrational band at 1749 cm^−1^ is assigned to v(C=O) of the ester group of ASPH (Figure 7) and at 1679 cm^−1^ to the v_as_(COO-) of ASPH [13]. The v_s_(COO-) of ASPH was not observed in the ATR-IR spectrum of the micelle since the vibration band is overlapped with those of SLS. The other intensive peak at 1606 cm^−1^ of **SLS@ASPH** was attributed to the ν(C=C) vibration of the aromatic ring [19]. No shift was observed between the vibrational bands of the ingredients and the corresponding ones in the micelle, suggesting no interactions between them. Therefore, the formation of the **SLS@ASPH** was concluded by vibrational spectroscopy.

Vibrational spectroscopy is a powerful tool in the investigation of structural changes at the molecular level in the short-range order. The ATR-FT-IR spectra of **SLS@ASPH**, ASPH, and SLS presented in Figure 7 further support the proposed structural model. In order to clarify whether the final product **SLS@ASPH** micelle is not composite material, the linear combination of the SLS and ASPH vibrational spectra was determined to be identical to the recorded spectrum of the **SLS@ASPH**.

Almost all the characteristic bands of the SLS and ASPH appeared in the spectrum of the **SLS@ASPH** micelle. We only observed differences in the relative intensities between the spectrum of **SLS@ASPH** and the spectra corresponding to the SLS and ASPH compounds. This is reasonable considering that the molecular structures of SLS and ASPH are retained in the formation of the **SLS@ASPH** complex. Furthermore, in the low-frequency region attributed mainly to bending modes, the relative intensity changes were drastic, while the frequencies of some bands exhibited a blue shift. This is expected for specific modes since the inter-atomic distance is reduced due to geometric restrictions, and thus, the energy of the vibration increases.

**Figure 8:**
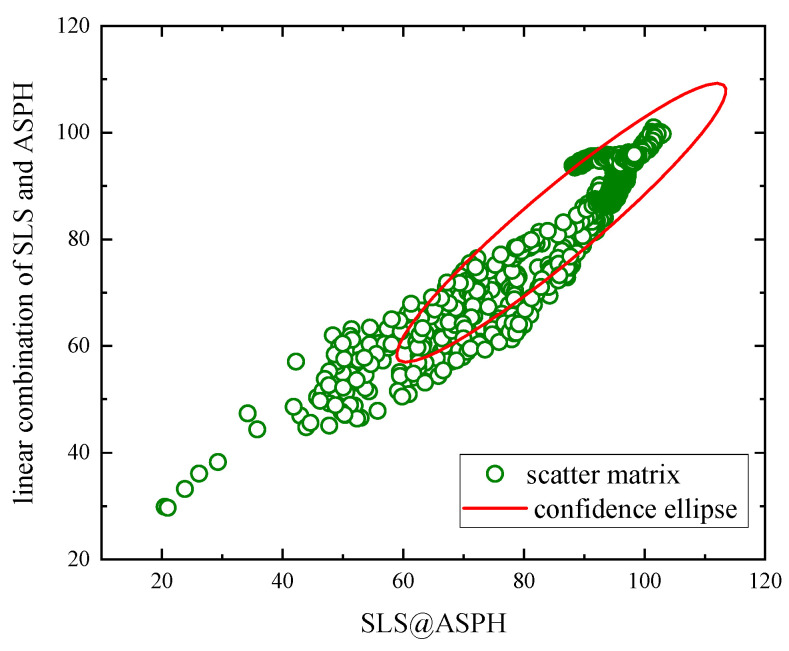
Confidence ellipse calculated from Pearson-type correlations for the SLS@ASPH complex in the solid state and the linear combination of the SLS and ASPH vibrational spectra.

In an effort to exclude the possibility of the solid residual after drying to be just a mixture of the two compounds, we performed correlation analysis between the spectrum of **SLS@ASPH** and the linear combination of the SLS and ASPH individual vibrational spectra. The results are presented in Figure 8.

*(b)*  *Solution state:*

**Figure 9:**
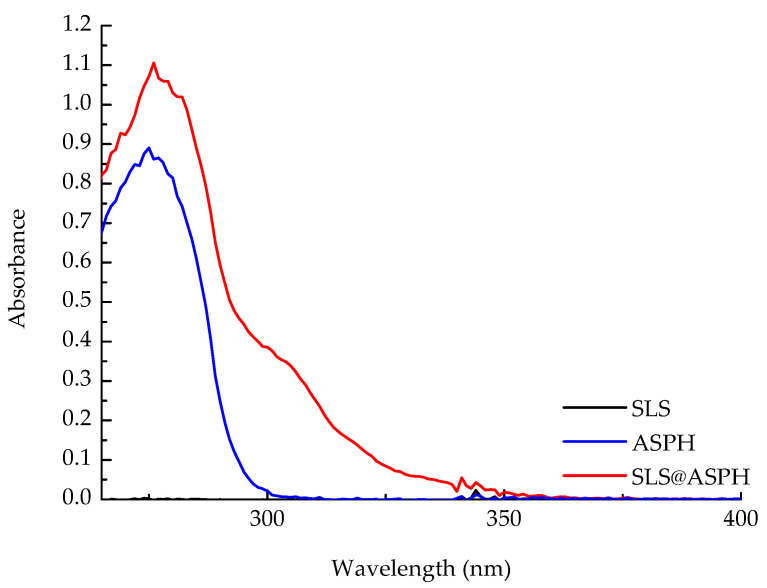
UV spectra of a DMSO solution of **SLS@ASPH** (2.7 mg/mL) and ASPH or SLS (0.30 mg/mL).

*Electronic absorption spectroscopy*: A DMSO solution of **SLS@ASPH** (2.7 mg/mL) and ASPH or SLS (0.30 mg/mL) was prepared, and the UV spectra were recorded (Figure 9). Since no electronic transition exists from the σ bonds of SLS, the absorption at 275 nm (Figure 9) should be attributed to the intra-ligand electron transition π* ← π of aspirin. By using the molar extension coefficient of ASPH at 275 nm (1070 L⋅mol^−1^⋅cm^−1^), the concentration of ASPH found in **SLS@ASPH** was 13.4% *w*/*w*. The content of ASPH in **SLS@ASPH** calculated by XRF spectroscopies was 11.11% (see XRF studies).

**Figure 10:**
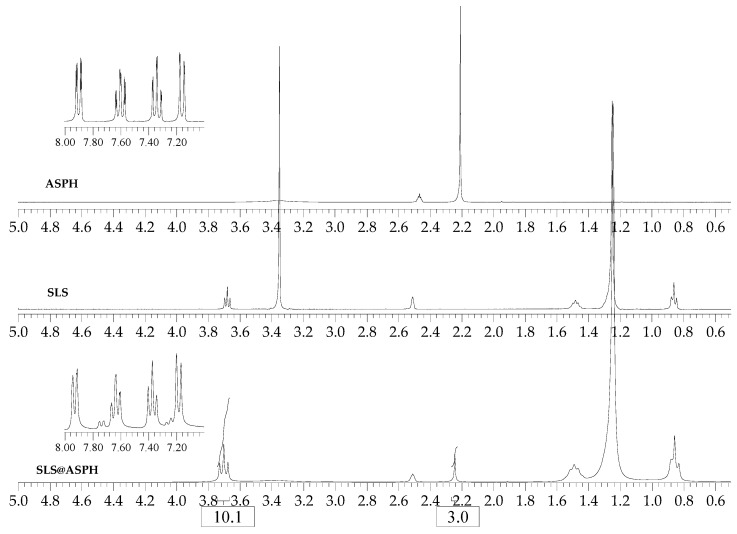
^1^H-NMR spectra of ASPH, SLS, and **SLS@ASPH** in DMSO-*d*_6_.

*Nuclear magnetic resonance (^1^H-NMR) spectroscopy*: The ^1^H NMR spectra of **SLS@ASPH** and of ligands were recorded (Figure 10). The resonance signals of the (H[-CH_2_^α’^-SO_3_-]) group of SLS at 3.70–3.66 ppm are slightly shifted in the spectrum of **SLS@ASPH** (3.79–3.69 ppm) [12,20]. The multiple resonance signals at 0.88–0.84 ppm in the spectrum of SLS are assigned to the protons of the [(-CH_3_)^ω’^] group of the SLS. The signals at 1.25 ppm and 1.50–1.47 ppm are attributed to the [(-CH_2_-)^10^] protons of SLS. The above signals were observed in the same frequency in the spectrum of **SLS@ASPH** (Figure 10).

The ^1^H NMR spectrum of ASPH is dominated by resonance signals at 7.92–7.88 (d, H^b^), 7.63–7.57 (t, H^d^), 7.37–7.31 (t, H^c^), 7.22–7.10 (d, H^e^), and 2.21 (s, H^g^[CH_3_CO-]) ppm [21]. The corresponding resonance signals of **SLS@ASPH** are slightly shifted at 7.94–7.91 (d, H^b^), 7.67–7.60 (t, H^d^), 7.40–7.34 (t, H^c^), and 7.20–7.17 (d, H^e^) ppm. The presence of the ASPH and SLS signals in the spectrum of **SLS@ASPH** indicates the encapsulation of the ASPH in the micelle. Moreover, due to integration, the signals of the 3 H^g^ [CH_3_CO-] protons correspond to 10.1 protons for the H^a’^[-CH_2_^α’^-SO_3_-]. Since 3 H^g^ [CH_3_CO-] corresponds to 1 ASPH and 10.1 protons for the H^a’^[-CH_2_^α’^-SO_3_-] to 5 SLS molecules, the SLS/ASPH = 5/1 molar ratio in **SLS@ASPH** was confirmed. This leads to a concentration of 12.5% *w*/*w* ASPH in **SLS@ASPH**. This value is in accordance with those determined by the XRF (11.11% *w*/*w*) and UV-Vis (13.4% *w*/*w*) spectroscopies (see XRF and UV-Vis spectroscopies).

No interaction between ASPH and SLS was detected from the spectroscopic characterization when the **SLS@ASPH** was formed, suggesting non-composite material formation. The properties of the components were therefore preserved. The 1:5 ASPH:SLS molar ratio in the micelle was confirmed in the solid state by XRF and in the solution by UV-Vis spectroscopy and NMR, confirming the retention of the micelle in both a solid and a solution. 

### 2.4. The Inhibitory Activity of SLS@ASPH towards Lipoxygenase (LOX)

Lipoxygenase (LOX) is an enzyme that catalyzes the formation of hydroperoxides from polyunsaturated fatty acids such as linoleic acid and arachidonic acid. LOX can be expressed in epithelial cells that display a variety of physiological functions, including inflammation, skin disorders, etc. [22]. LOX has been suspected of playing a role in cutaneous homeostasis [23]. Inhibitors of the enzyme 5-LOX have been shown to curtail inflammation in a different model [23]. The enzyme 15-LOX-2 discovered in the skin in humans shows higher similarity to 15-LOX (glycine max (soybean)) [22].

**Figure 11:**
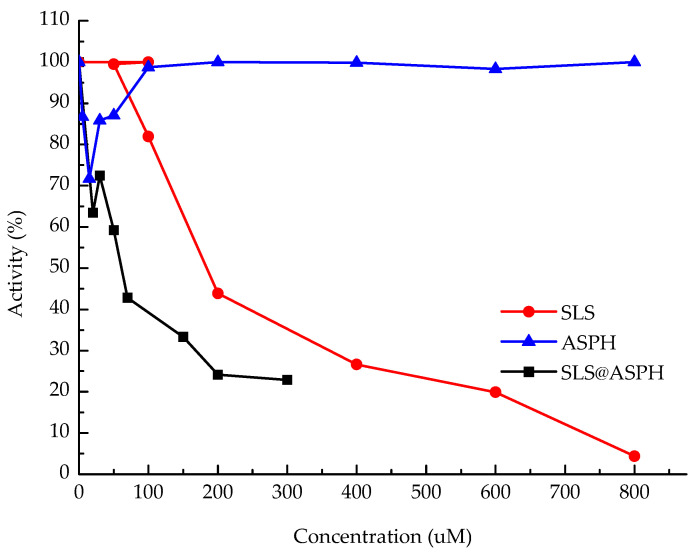
The inhibitory effect against LOX activity caused by **SLS@ASPH**, SLS, and ASPH.

The influence of **SLS@ASPH** and its ingredients (SLS and ASPH) on the oxidation of linoleic acid by 15-LOX was studied by UV spectroscopy. The degree of LOX activity (A, %) was determined [24]. Figure 11 compares the inhibitory effect of **SLS@ASPH** and its ingredients. The IC_50_ values of the agents towards LOX activity are 74.8 μΜ (in respect to the ASPH) for **SLS@ASPH** and 237 μΜ in the case of SLS, while ASPH is a non-LOX inhibitor [25]. Furthermore, ASPH showed no inhibitory activity up to the tested concentration of 800 μΜ (Figure 11). For this case, a DMSO solution of ASPH was used due to the low solubility of ASPH in water. Furthermore, to the best of our knowledge, the SLS inhibitory activity towards LOX is reported here for the first time. 

### 2.5. In Vitro Toxicity against Immortalized Human Keratinocytes (HaCaT) Cells

The HaCaT cell line is useful for the testing of preventive and therapeutic strategies for skin [26], and it can represent a model for studying cellular pharmacokinetics of drugs on keratinocytes [27]. The toxic activity of the micelle and its ingredients was tested against HaCaT cells for 48 hrs according to sulforhodamine B (SRB) assay. The IC_50_ value for **SLS@ASPH** is 2.1 ± 0.1 μΜ (in respect to ASPH) against HaCaT, while the corresponding values for SLS and ASPH (in DMSO) are 64.9 ± 3.5 μΜ and >300 μΜ, respectively. Therefore, the encapsulation of ASPH in SLS increases the toxicity activity of the micelles in water in respect to its components due to their synergy.

## 3. Materials and Methods

### 3.1. Materials and Instruments

All solvents used were of reagent grade. SLS (Sigma-Aldrich, St. Louis, MO, USA, product of Spain,) and aspirin (Sigma-Aldrich, St. Louis, MO, USA, product of Spain) were used with no further purification. Melting points were measured in open tubes with a Stuart Scientific apparatus and were uncorrected. IR spectra in the region of 4000–370 cm^−1^ were obtained using a Cary 670 FTIR spectrometer (Agilent Technologies Santa Clara, CA, USA). The ^1^H-NMR spectra were recorded on a Bruker AC 400 MHz FT-NMR instrument in a DMSO-*d*_6_ solution (Bruker GmbH & Co. 28359 Bremen, Germany). A UV-1600 PC series spectrophotometer of VWR international GmbH, Darmstadt, Germany, was used to obtain electronic absorption spectra.

### 3.2. Determination of Critical Micelle Concentration (CMC) by Solutions Density Measurements

A DMA pycnometer (Anton Paar Germany GmbH, Scharnhausen, Ostfildern, Germany) was used for the determination of the solutions density at a select temperature. The accuracy of the measured density was ±0.0001 g/mL. The kinematic viscosities of the solutions were measured by means of an Ubbelohde-type viscometer that was temperature-controlled to within ±0.1 °C. From the obtained densities and kinematic viscosity values, we calculated the dynamic viscosity of all solutions with an accuracy of ±1%.

Adiabatic compressibility for all solutions was calculated from the mass density and the corresponding sound speed as $\kappa_{s}={\left(\rho u_{s}^{2}\right)}^{-1}$. 

Surface tension measurements were performed using a torsion balance fitted with a platinum ring (Krüss, model K8).

All measurements were carried out at 20 °C under isobaric conditions. More details concerning the experimental protocols used can be found elsewhere [28,29].

### 3.3. Determination of CMC via Ultrasonic Velocity

The solution was inserted into a temperature-controlled (to within ±0.1 °C) acoustic cell. The amplitude of the ultrasonic wave was continuously monitored by a digital oscilloscope (Tektronix, TBS 1202B, Tektronix Inc. Beaverton, OR 97077, USA) while being passed multiple times through the acoustic cell. A set of two identical broadband transducers (Olympus V111) operating at a frequency of 10 MHz was used as the transmitter and receiver of the ultrasound for the complete set of velocity measurements by applying the overlapping pulse-echo methodology [15,16]. A frequency generator (TTi, TGP3151) sent the appropriate electric pulse to the transmitter piezoelectric element. This experimental procedure permitted accurate velocity measurements in the liquid with an experimental error less than ±0.01%.

**Figure 12:**
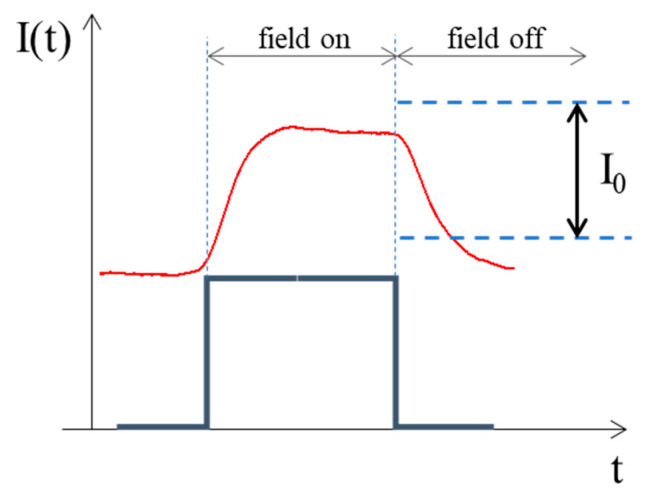
The square-shaped ultrasonic pulse applied to the system (thick blue solid line) and the corresponding response of the system (thinner red line) in the presence (field on) and in the absence (field off) of the ultrasonic field.

The application of an ultrasonic field of a specific frequency on an isotropic liquid induces differences in the refractive index Δ*n* related to the *n*_┴_ and *n*_//_ that correspond to the refractive indexes, which are perpendicular and parallel to the direction of the sound wave propagation, respectively. The experimental setup for the transient acoustically induced birefringence was described in detail in [11,17]. In brief, an acousto-optical cell made of glass was filled completely with the liquid sample and thermo-stated at the desired temperature. The sample container was sealed, eliminating potential directional flow or liquid sharing. A piezoelectric element with a fundamental frequency near 1 MHz was used to deliver the applied square electric pulse into the liquid as an ultrasonic wave. The liquid was perfectly and evenly exposed to the applied acoustic field. In the perpendicular direction with respect to the sound wave propagation, a linearly polarized electromagnetic field (He-Ne laser, Melles-Griot, λ = 632.8 nm, power = 5 mW) was applied. The optical and the acoustic path lengths were fixed at 1 cm. The light source was used as a probe for the estimation of the birefringence signal induced after the application of the ultrasonic field. The cell filled with solution was placed between a polarizer and a quarter-wave plate that was oriented in cross-polarization relative to the polarizer. A photodiode with fast response was utilized to monitor the induced birefringence signal in an oscilloscope. The pulse length was always adjusted to be long enough to reach a steady value of the ultrasonically induced birefringence, as presented in Figure 12.

It seemed that after the application of the acoustic field, the system exhibited a delay in reaching its maximum birefringence signal, the so-called stationary birefringence. The observed relaxational behavior of the chemical system was reflected in the birefringence signal and was related to the non-spontaneous orientation process of the building units constituting the system and finally approached a constant value (plateau), namely the maximum (stationary) birefringence. The specific increasing rate was affected by the nature of the structural species. On the other hand, when the ultrasonic field was off, a specific decay rate was exhibited that reflected the reverse, although spontaneous, disorientation relaxation process of the molecules. Therefore, two distinct processes with not necessarily the same rise and decay rates were demonstrated by the system during the presence and absence of the ultrasonic field. In this work, we restricted our analysis to the decay relaxation process due to its spontaneous character.

### 3.4. Formation of SLS@ASPH

A total of 0.091 g (0.5 mmol) of aspirin was added to 0.63 g (2.2 mmol) SLS in 7 mL of a water solution at 37 °C. The molar ratio for [SLS]:[ASPH] was 5/1. The solution was stirred for a half hour, filtered off, and concentrated to dryness.

For ***SLS@ASPH***, the yield was 30%; melting points were 189–193 °C; IRs (cm^−1^) were 3464 w, 2964 m, 2918 vs, 2851 s, 1749 m, 1681 m, 1604 m, 1465 m, 1261 vs, 1080 s, 971 m, 916 m, 833 m, 754 m, 632 m, and 590 s; ^1^H-NMR (ppm) in DMSO-*d*_6_ were 7.94–7.91 (d, H^d^ of ASPH), 7.67–7.60 (t, H^f^ of ASPH), 7.40–7.34(t, H^e^ of ASPH), 7.20–7.17 (d, H^g^ of ASPH), 3.79–3.69 (t, H[-CH_2_^α’^-SO_3_-] of SLS), 1.25 (s, (-CH_2_-)^10^ of SLS), and 1.50–1.47 (m, (-CH_2_-)^10^ of SLS); and UV (DMSO or H_2_O) was λ_max_ = 276 nm.

### 3.5. The Inhibitory Activity of SLS@ASPH towards Lipoxygenase (LOX)

This study was performed as previously reported [24]. Briefly, the buffer used for the LOX experiments was 0.2 M boric acid at pH 9. Then, 0.1 mol boric acid (H_3_BO_3_, 6.18 g) was added to 300 mL distilled water. The pH was adjusted to 9 with 50% *w*/*v* NaOH. Finally, the solution was diluted to 500 mL. A linoleic acid substrate solution was prepared as described below: 0.05 mL of linoleic acid was dissolved in 0.05 mL 95% ethanol in a volumetric flask (50 mL). The appropriate volume of H_2_O was gradually added to the flask. Then, 5 mL of the prepared solution was added to 30 mL of the borate buffer. The LOX enzyme solution was a solution of 10,000 units of enzyme for each cubic centimeter of borate buffer prepared in a cold ice bath. A total of 500 units for every 3 mL of reaction mixture was used in every experiment. A unit of lipoxygenase causes an increase in absorption at 234 nm, equal to 0.001 per minute [24].

The enzyme activity was monitored by UV analysis. The substrate concentration was kept constant (0.3 mM), while the amounts of buffer and inhibitor solutions varied according to the final concentration of inhibitor needed (0–800 μM). The enzyme solution (0.05 mL) was added to a cuvette containing 2 mL linoleic acid solution and the appropriate amounts of buffer (0.950–0.926 mL) and inhibitor (0–0.024 mL) DMSO solution (0.1 M) in a thermostatic water bath at 25 °C. The maximum percentage of DMSO in the buffer solution was 0.8% *v*/*v*. The total experiment volume was 3 mL. There was no pre-incubation time for the enzyme with the inhibitor solution. The activity of the enzyme was determined by monitoring the increase in the absorption caused by the oxidation of linoleic acid at 234 nm and 25 °C (ɛ = 25,000 M^−1^ cm^−1^) [24].

### 3.6. Sulforhodamine B Assay

These studies were performed in accordance with a previously reported method [10,13]. Briefly, HaCaT cells were seeded in a 96-well plate at a density of 8000 cells per well, and after 24 h of cell incubation, the compounds were added in the concentration range of 0.5–6 μM for **SLS@ASPH**, 50–300 μΜ for ASPH, and 20–140 μΜ for SLS. HaCaT cells were exposed to the compounds for a period of 48 h. The immortalized human skin keratinocyte cell line (HaCaT) was kindly offered by Prof. Dr. P. Pappas, Department of Pharmacology, Medical School, University of Ioannina, 45110 Ioannina, Greece.

## 4. Conclusions

In conclusion, the water-soluble micelle **SLS@ASPH** was prepared by aspirin and sodium lauryl sulfate. The encapsulation of ASPH in the SLS micelle with nuclearity equal to 1:5 enhances its solubility in water significantly (Figure 5). This reflects in the inhibitory activity of **SLS@ASPH** against LOX activity, which is 3.5-fold stronger than that of free SLS. The inhibitory activity of ASPH against LOX is negligible as expected since ASPH is a well-known COX inhibitor. However, the micellization of ASPH in SLS increases this activity of SLS.

## Data Availability

Not applicable.

## Supplementary Materials

The following supporting information can be downloaded at: https://www.mdpi.com/article/10.3390/ijms242015152/s1. References [30,31,32] are cited in the supplementary materials.
