# Supplementary material for: Non-Steroidal Anti-Inflammatory Drugs Loaded to Micelles for the Modulation of Their Water Solubility
Source: Int J Mol Sci. 2023 Oct 13;24(20):15152. doi: 10.3390/ijms242015152 (PMC10607354; doi:10.3390/ijms242015152)
Supplement: Supplementary file 1 [file ijms-24-15152-s001.zip › ijms-2596552-SI.pdf]

International Journal of *Molecular Sciences*

MDPI

Article

Supplementary Information

Non-Steroidal Anti-Inflammatory Drugs Loaded to Micelles for the Modulation of Their Water Solubility

Christina N. Banti [1], Angelos G. Kalampounias [2,3,*] and Sotiris K. Hadjikakou [1,3,*]

[1] Inorganic Chemistry Laboratory, Department of Chemistry, University of Ioannina, 45110 Ioannina, Greece; cbanti@uoi.gr
[2] Physical Chemistry Laboratory, Department of Chemistry, University of Ioannina, 45110 Ioannina, Greece
[3] Institute of Materials Science and Computing, University Research Center of Ioannina (URCI), 45110 Ioannina, Greece
* Correspondence: akalamp@uoi.gr (A.G.K.); shadjika@uoi.gr (S.K.H.); Tel.: +30-26510-08374 (S.K.H.)

Aggregation process.

Upon dissolution of surfactant into water, the clathrate structure of water is interrupted by the presence of hydrophobic groups that finally lead to an increase of the free energy. The molecules of surfactant concentrate at surfaces aiming to compensate the free energy increase through directing their hydrophobic groups away from the water. The free energy minimization leads to the clustering of the surface-active molecules forming micelles. During this aggregation process, namely the micellization, the hydrophobic groups are directed towards the interior of the cluster and the hydrophilic groups are directed on the opposite direction that is the water side. This process is dynamical in nature and facilitates the confinement of the surfactant molecules to micelle through lowering of their degrees of freedom. These molecules interact via electrostatic repulsion forces originating from the presence of similarly charged surfactant molecules. On the other hand, hydrophobic and van der Waals forces enhance the aggregation. Thus, the successful formation of micelles is controlled by the balance between favoring and opposing factors. The surfactant association through hydrophobic bonding is succeeded by the domination of hydrophobic and van der Waals forces over kinetic energy of the molecules and electrostatic repulsion forces.

The kinetics of micellization provides information concerning the size of the formed micelles and their polydispersity. The fast relaxation time τ_1 provided by ultrasonic relaxation measurements is a function of the surfactant concentration and can be estimated as [Suppl_Refs 1,2]:

$$\frac{1}{\tau_1} = 2\pi f_r = \frac{k_-}{\sigma^2} + \frac{k_-}{n}\left(\frac{C}{cmc} - 1\right) \quad \text{(s1)}$$

with k_- representing the backward (mean dissociative) rate constant. Parameter σ^2 stand for the micellar distribution or the micellar polydispersity and it is a non-stoichiometric parameter. The backward to forward rate constant ratio is almost equal to the critical micelle concentration [Suppl_Ref 3]. Equation (s1) predicts a linear dependence of the reciprocal relaxation time $1/\tau_1$ on the reduced critical concentration $\left(\frac{C}{cmc} - 1\right)$. Thus, the aggregation number, the backward rate constant and the micellar distribution can be determined from the slope and the intercept of this graph. Fig. S1 represents the dependence of the reciprocal fast relaxation time on the reduced critical micelle concentration in a concentration region above cmc. A clear linear dependency is detected between $1/\tau_1$ and $\left(\frac{C}{cmc} - 1\right)$. The so-obtained backward and forward rate constants are k_-=1.92×10^7 s^{-1} and k_+=1.2×10^{10} M^{-1}s^{-1}, respectively. The micellar polydispersity that is the half-width of the Gaussian distribution curve of micellar population, was found equal to σ =1.69.

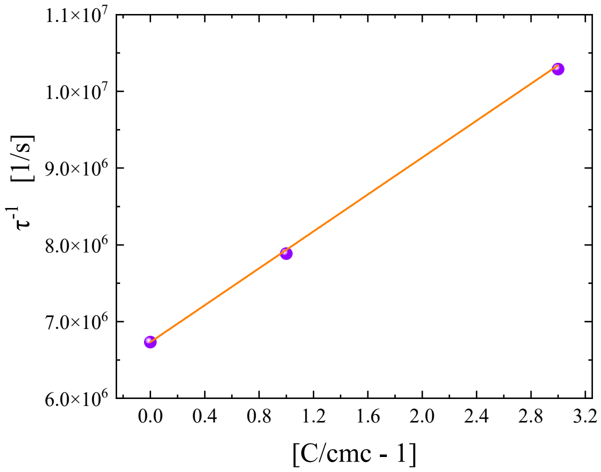

Figure S1. Inversed relaxation time as a function of the reduced critical micelle concentration at 20°C.

Supplementary References

[1] Aniansson, E.A.G.; Wall, S.N. Kinetics of step-wise micelle association. *J. Phys. Chem.* **1974**, *78*, 1024–1030; Correction and improvement in *J. Phys. Chem.* **1975**, *79*, 857–858.

[2] Aniansson, E.A.G.; Wall, S.N.; Almgren, M.; Hoffmann, H.; Kielmann, I.; Ulbricht, W.; Zana, R.; Lang, J.; Tondre, C. Theory of the kinetics of micellar equilibria and quantitative interpretation of chemical relaxation studies of micellar solutions of ionic surfactants. *J. Phys. Chem.* **1976**, *80*, 905.

[3] Thomason, M.A.; Bloor, D.M.; Wyn-Jones, E. Ultrasonic relaxation and micelle formation in solutions of cetylpyridinium chloride in formamide. *Langmuir*, **1992**, *8*, 2107.
